# Towards the Experimentally-Informed *In Silico* Nozzle Design Optimization for Extrusion-Based Bioprinting of Shear-Thinning Hydrogels

**DOI:** 10.3389/fbioe.2021.701778

**Published:** 2021-08-06

**Authors:** Esther Reina-Romo, Sourav Mandal, Paulo Amorim, Veerle Bloemen, Eleonora Ferraris, Liesbet Geris

**Affiliations:** ^1^ Department of Mechanical Engineering and Manufacturing, University of Seville, Seville, Spain; ^2^ Biomechanics Research Unit, GIGA In Silico Medicine, Université de Liège, Liege, Belgium; ^3^ Prometheus, The Division of Skeletal Tissue Engineering, KU Leuven, Leuven, Belgium; ^4^ Materials Technology TC, Campus Group T, KU Leuven, Leuven, Belgium; ^5^ Department of Mechanical Engineering, Campus de Nayer, KU Leuven, Leuven, Belgium; ^6^ Skeletal Biology and Engineering Research Center, KU Leuven, Leuven, Belgium; ^7^ Biomechanics Section, Department of Mechanical Engineering, KU Leuven , Leuven, Belgium

**Keywords:** power law fluid model, computational modeling, computational fluid dynamics, machine learning, bioink, cell viability, design of experiements, Gaussian process

## Abstract

Research in bioprinting is booming due to its potential in addressing several manufacturing challenges in regenerative medicine. However, there are still many hurdles to overcome to guarantee cell survival and good printability. For the 3D extrusion-based bioprinting, cell viability is amongst one of the lowest of all the bioprinting techniques and is strongly influenced by various factors including the shear stress in the print nozzle. The goal of this study is to quantify, by means of *in silico* modeling, the mechanical environment experienced by the bioink during the printing process. Two ubiquitous nozzle shapes, conical and blunted, were considered, as well as three common hydrogels with material properties spanning from almost Newtonian to highly shear-thinning materials following the power-law behavior: Alginate-Gelatin, Alginate and PF127. Comprehensive *in silico* testing of all combinations of nozzle geometry variations and hydrogels was achieved by combining a design of experiments approach (DoE) with a computational fluid dynamics (CFD) of the printing process, analyzed through a machine learning approach named Gaussian Process. Available experimental results were used to validate the CFD model and justify the use of shear stress as a surrogate for cell survival in this study. The lower and middle nozzle radius, lower nozzle length and the material properties, alone and combined, were identified as the major influencing factors affecting shear stress, and therefore cell viability, during printing. These results were successfully compared with those of reported experiments testing viability for different nozzle geometry parameters under constant flow rate or constant pressure. The *in silico* 3D bioprinting platform developed in this study offers the potential to assist and accelerate further development of 3D bioprinting.

## Introduction

Bioprinting is a research-intensive field within regenerative medicine combining additive manufacturing technologies and tissue engineering concepts for reproducing functional organs and complex living tissues in the laboratory (Murphy and Atala, 2014; Ji and Guvendiren, 2017; Moroni et al., 2018b; Chen et al., 2021). However, there are still many challenges in order to guarantee cell survival and good printability (Bishop et al., 2017; Moroni et al., 2018a; Ong et al., 2018; Sánchez et al., 2020). The three predominant techniques used for bioprinting are inkjet printing (or drop-by-drop bioprinting), extrusion bioprinting and laser-induced patterning (Bishop et al., 2017; Cidonio et al., 2019; De Moor et al., 2020). When using a bioink, the main goal of these techniques is either to position different cell types in desired locations or to induce progenitor cells to differentiate into the desired type at specific locations (Derby, 2012; Skardal, 2018). Extrusion-based bioprinting is one of the most widely used technique in current research, because of it is simplicity and ability to print a broad array of biocompatible materials and to deposit variable and high cell densities at specific locations in the three-dimensional (3D) space (Gillispie et al., 2020). The set-up is also highly adaptable and is ideally suited for the fabrication of large and complex tissue constructs since it allows building up 3D structures according to a layer-by-layer approach (Mandrycky et al., 2016). On the other hand, the development of a bioink with suitable rheology and printability response is not straightforward. In addition, cell viability is amongst the lowest across all the bioprinting techniques.

Despite the extensive experimental work carried out on extrusion-based 3D bioprinting, a comprehensive view on the individual and combined effects of various parameters on cell fate is not straightforward to derive (Nair et al., 2009; Murphy and Atala, 2014; Mandrycky et al., 2016; Paxton et al., 2017; Moroni et al., 2018a). Cell viability has been reported to be as low as 40% (Mandrycky et al., 2016) and shear stress has been identified as one of the main causes (Gillispie et al., 2020). Fluid shear stress has been shown to influence cell functionality *in vivo* (Wittkowske et al., 2016) and *in vitro* (Pedersen et al., 2016; Silvani et al., 2021). Probing into the maintenance of cell viability and functionality, many studies have made it clear that higher shear stresses (beyond physiological ranges) almost always lead to lower cell viability and altered functionality (Li et al., 2009; Nair et al., 2009; Ozbolat, 2016). Blaeser *et al.* found that shear stress should be controlled within 5 kPa to have more than 90% cell survival for mouse fibroblasts in a microvalve-based printing process (Blaeser et al., 2016). Higher dispensing pressure can allow ejecting highly viscous bioinks, but this could increase the shear stress, which reduces cell viability *in vitro*. A few studies observed only a small or even a non-discernible change in viability in relation to increased shear stress during printing (Khalil and Sun, 2009). Shear stress is a factor that cannot be directly measured during experiments, however the level of shear stress is directly related to different material and bioprinting parameters, such as bioink viscosity, extrusion pressure, flow rate and also nozzle geometry (shape and size) (Nair et al., 2009; Billiet et al., 2014; Blaeser et al., 2016; Sánchez et al., 2020). Dispensing pressure and flow rate of the dispensed material also directly affect the print speed as well as scaffold attributes such as printed strut thickness, macroscopic pore size and porosity of the scaffolds formed, and hence their mechanical properties (Li et al., 2011; Cidonio et al., 2019). In addition, the material properties of the hydrogel used to encapsulate the cells, such as the shear-thinning behavior, have an important influence on the printability of the bioink. The experimental results reported in the literature follow different printing protocols and material characterization strategies, making direct comparison difficult to perform. Further work is needed to improve and standardize the currently used printability measurements and to move towards a more comprehensive view and reporting of printability and 3D printing (Gillispie et al., 2020).

Computational modeling (*in silico*) allows obtaining a more comprehensive view of the physical phenomena encountered in diverse tissue engineering applications (Reina-Romo et al., 2019; Mehrian et al., 2020; Mukherjee et al., 2020). Models of extrusion-based 3D bioprinting have evolved from analytical models requiring simplifying assumptions on nozzle geometry to computational fluid dynamics (CFD) models that can simulate complex flow behavior and complex nozzle geometries (Emmermacher et al., 2020). *In silico* models enable the visualization and quantification of the mechano-chemical micro-environment of the cells during the bioprinting process under various protocols. Li et al. developed an analytical computational model to investigate cell damage with different needle geometries and found that at equivalent flow rates, cell damage in a conical nozzle is lower than in a blunted one (Li et al., 2009, Li et al., 2011). The optimization of computer-controlled process parameters has been addressed in a handful of experimental studies. The increase in computational power and the rapid development of various algorithms are making *in silico* models ideally suited to address this optimization challenge when combined with various data analysis techniques such as machine learning. A variety of methods are available to account for variations in design parameter data and to quantify the importance of each parameter (Hamby, 1994). The design of experiments (DoE) is one such method for studying the influence of the input parameters on physical experiments and computational models, through optimisation and outcome prediction in the whole parameter space, resulting in reduced cost, time and variability (Montgomery, 2017; Dellaquila et al., 2020). The results of the DoE can be analyzed through several methods, depending on the design and the goal of the analysis. Statistical tools such as Analysis of variance (ANOVA) are conventionally used for analyzing factorial design outcomes (Gramacy and Lee, 2009). In case of more complex space-filling designs, machine learning algorithms, (such as “Gaussian process”, GP) are more appropriate to estimate the importance of a parameter and the exact effect of varying a parameter on the outcome of the model (Gramacy, 2007; Gramacy and Lee, 2009). Unlike an ANOVA-type of analysis, Gaussian processes not only estimate the importance of individual parameters, but also the influence of the parameters on the outcome of a model (Gramacy, 2007).

In this study, we developed an *in silico* framework to assess and quantify the effect of the nozzle geometry, printing pressure and material properties on the shear stress and related cell viability during extrusion-based bioprinting. Two different nozzle configurations are investigated: conical and blunted (or cylindrical). Three commonly used hydrogel materials are incorporated: Alginate-Gelatin, Alginate and PF127 (Sánchez et al., 2020; Roche et al., 2021). A DoE approach is used to screen 200 different combinations of geometrical parameters (nozzle design) per geometry and per hydrogel. For each design, the shear stresses arising in the nozzle during extrusion are computed using a CFD model. Comparison between the *in silico* results for specific studies reported in the literature allows validating of the computational model and the use of shear stress as a surrogate for cell viability. Machine learning (Gaussian Process) is used to analyze the (*in silico*) screening data and identify the geometrical parameters that have the highest influence on the shear stress and related cell viability. This revealed the important parameters to be considered as well as insight into the complex combinatorial effect of nozzle geometry and hydrogel material properties.

## Materials and Methods

### Material Properties

An incompressible, non-Newtonian fluid material model (at constant temperature) was used to simulate the flow of the three hydrogels considered in this study: Alginate-Gelatin or AlgGel (4%w/v Alg with 20%w/v Gel, mixed in 1:1), Alginate (12%w/v with 1%w/v CaCl_2_) and pluronic F127 or PF127 (25%w/v) (Rezende et al., 2009; Chung et al., 2013; Lee and Yeong, 2015; Suntornnond et al., 2016; Paxton et al., 2017; Roche et al., 2021). PF127 is a biologically inert material compared to the other two which support cell adhesion, however it has been utilized frequently for its shear thinning capability as a sacrificial or support material in conjunction with other hydrogels, and successful modification of PF127 to support cell adhesion is also reported (Gillispie et al., 2020). There are several material models to capture the behavior of the viscosity, and more specifically the shear-thinning behavior of the hydrogel. In this study, the rheological model used to simulate the flow behavior is the power-law relationship, ubiquitous in the 3D bioprinting literature:

$$\eta =K\cdot {\dot{\gamma }}^{n-1}$$ (1)

$$\tau =\eta \dot{\gamma }=K\cdot {\dot{\gamma }}^{n}$$ (2)

where, η is the viscosity, $\dot{\gamma }$ is the shear rate, K is the consistency coefficient and *n* is the power-law or flow behavior index (Chhabra and Richardson, 2008). Newtonian fluids are power-law fluids with a behavior index *n* of 1, however materials with *n* > 0.8 practically behave as Newtonian fluid (Chhabra and Richardson, 2008). The densities, consistency coefficient and power-law indices, as well as the common printing pressures are reviewed in Table 1. The consistency coefficients and power-law indices were empirically obtained from rheological data fitting (see Supplementary Figure S1). The data was collected from several sources in the literature, with varying material concentrations and printing pressures (Table 1). The parameters used in this study were chosen within this range, following Paxton et al. (2017).

**TABLE 1:** Range of material properties and common printing pressures of the hydrogels considered in this study. The values used in the present study are primarily from Paxton et al. (2017).

Material	Range	Density ρ (kg/m^3^)	Power-law index n (−)	Consistency coefficient K (Pa·s^n^)	Printing pressure P (kPa)	References
Pluronic F127 (PF127)	Literature	1,040	0.051–0.988	0.06–510	100–300	Jalaal et al. (2017); Paxton et al. (2017)
	Present study	1,000	0.127	406	200	
Alginate (Alg)	Literature	–	0.188–0.433	27.3–254	150–500	Rezende et al. (2009); Lee and Yeong, (2015); Paxton et al. (2017)
	Present study	1,000	0.335	55.7	340	
Alginate-Gelatin (AlgGel)	Literature	–	0.608	13.3	80–300	Wüst et al., (2015); Wüst et al., (2015); Paxton et al. (2017); Roche et al. (2021)
	Present study	1,000	0.608	13.3	80	

### Nozzle Geometry and Design of Experiments

**FIGURE 1:**
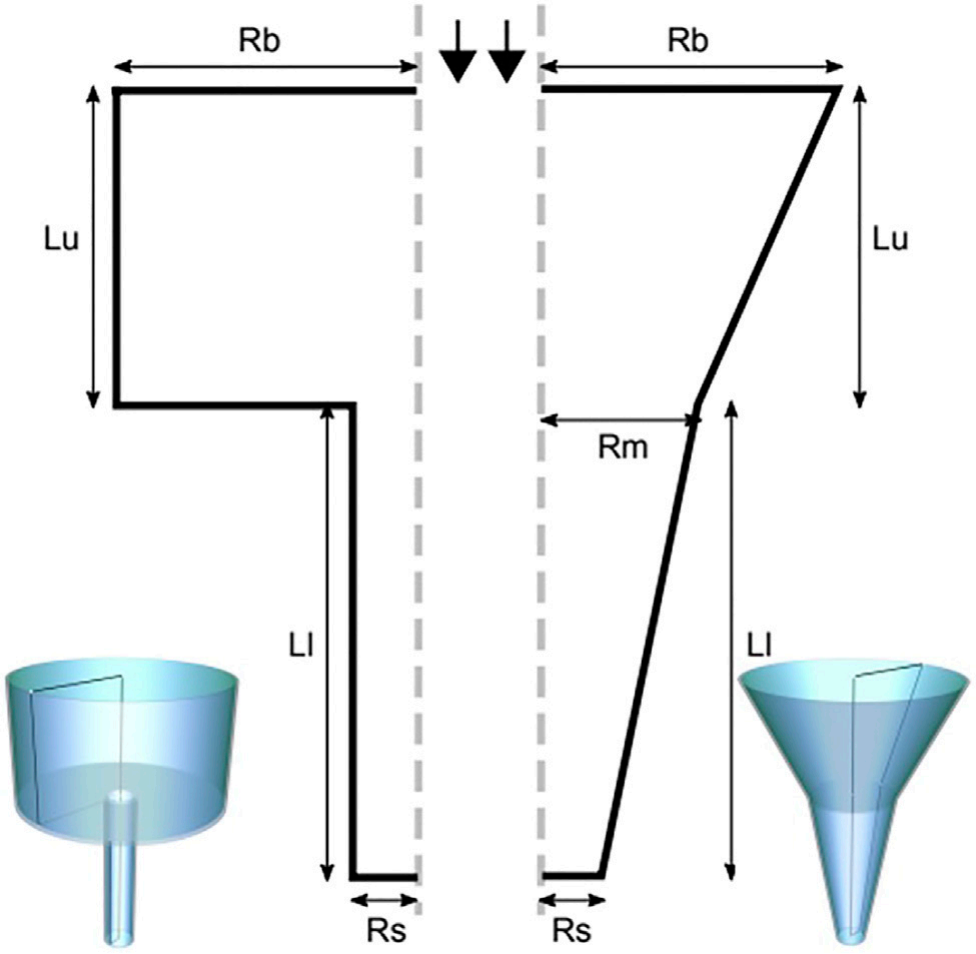
The blunted **(left)** and the conical **(right)** nozzle geometries, described by geometric parameters radius at the entrance (*R*_*big*_ or Rb) and the exit (*R*_*small*_ or Rs) of the nozzle, the inlet (*L*_*upper*_ or Lu) and the outlet (*L*_*lower*_ or Ll) nozzle lengths and the radius at the middle (*R*_*middle*_ or Rm, for the conical nozzle only).

The nozzle geometries incorporated in this study are the conical (tapered) and the blunted (cylindrical) shape, comprehensibly described by 5 geometrical parameters for the conical nozzle and 4 geometrical parameters for the blunted one, as indicated in Figure 1. For the blunted nozzle design these are: the radius at the entrance (*R*_*big*_) and the exit (*R*_*small*_) of the nozzle, the inlet (*L*_*upper*_) and outlet (*L*_*lower*_) nozzle lengths. For the conical nozzle design an extra parameter is required: the intermediate radius of the nozzle (*R*_*middle*_). CAD model design for illustration was done using FreeCAD (Riegel et al., 2001) and Inkscape (Oualline and Oualline, 2018).

In order to determine the importance of the aforementioned geometrical parameters as well as all possible combinations, a DoE was conducted. Within the DoE, each of the investigated parameters adopted different values within a given range. The chosen range of each geometrical parameter was adopted from the catalog of Nordson EFD optimum systems (Systems, 2018). Table 2 shows the geometrical parameters and experimental design ranges used.

**TABLE 2:** Range of the values of the geometrical parameters considered for the conical and blunted (cylindrical) nozzle designs.

Nozzle shape	Variable	Range (mm)
Conical	*R*_*small*_	0.05–0.42
	*R*_*middle*_	1–3.5
	*R*_*big*_	1.5–4.5
	*L*_*lower*_	4–20
	*L*_*upper*_	3–15
Blunted	*R*_*small*_	0.05–0.42
	*R*_*big*_	1.5–4.5
	*L*_*lower*_	4–20
	*L*_*upper*_	3–15

There are different methods to perform the sensitivity analysis by DoE such as the factorial designs, either full or fractional, the Taguchi’s Method or the space-filling designs, such as the Latin Hypercube Sampling or the uniform design (Isaksson et al., 2006; Myers et al., 2016). More details on these can be found in Montgomery (Montgomery, 2017), Myers and Montgomery (Myers et al., 2016) and (Saltelli et al., 2008). Firstly, a space-filling design, known as Latin Hypercube Sampling or Latin Hypercube Design (LHS or LHD) was employed to spread out the parameter combinations over the entire parameter space to be able to capture the full range of possible behaviors. The main advantage of this method is that it is computationally cheap to generate and can deal with a large number of parameters (McKay et al., 1979). Compared to a completely random Monte Carlo method for picking parameters in a given range, LHS divides the parameter space (the dimension of which is equal to the number of input parameters) into equal “bins”. Subsequently, it randomly picks values from each bin which leads to a better sampling, which is why it is referred to as “space-filling” (Gramacy, 2007; Gramacy and Lee, 2009). In this study, an LHD with 200 valid parameter combinations was generated, using “lhs” package in R (Carnell and Carnell, 2019). For the conical nozzle, parameter combinations with the entrance radius (*R*_*big*_) smaller than the intermediate radius (*R*_*middle*_) were excluded. Finally, each LHD contained 200 possible nozzle designs covering the entire design spectrum for all geometrical parameters. In total six different LHDs were performed, to analyze the three hydrogels (Alginate, PF127 and Alginate-Gelatin) for the two nozzle geometries (blunted and conical).

### Computational Fluid Dynamics Model

CFD fluid flow simulations were conducted in the open-source CFD software platform OpenFOAM (version 7) (Weller et al., 1998, 2019). The simulation domain consisted of the fluid (i.e. the hydrogel) inside the nozzle (Figure 1). The fluid was simulated considering a steady laminar flow (no turbulence) since laminar behavior is reported for non-Newtonian fluid in similar applications (Chhabra and Richardson, 2008). We assumed a simplified axisymmetric representation of the geometry with rotational symmetry along the long nozzle axis (Figure 1). The flow was simulated using the boundary conditions of a zero-wall velocity (no-slip condition) and constant printing pressure “Δ*p*” (net pressure difference between the inlet and the outlet) ranging between 80 and 340 kPa, depending on the material (Table 1). For a few simulations in the later part of the study, a constant input flow rate boundary condition was used in place of the “Δ*p*”.

A 2D axisymmetric mesh composed of hexahedral elements with prisms only along the axis of symmetry was used in all the cases. A convergence analysis was carried out to ensure the grid-independence of the results. From this, for all the models, the suitable grid cell size was determined as 0.005 mm in the radial direction (orthogonal to the flow direction) and 0.010 mm in the flow direction. Simulation results were validated by comparison with analytical results for simplified geometries and the commercial software package Ansys PolyFlow (Stolarski et al., 2018). For the analytical model from literature, the nozzle geometry was reduced to the lower part of the nozzle, especially for the blunted nozzle (Khalil and Sun, 2009; Rezende et al., 2009; Lee and Yeong, 2015; Paxton et al., 2017), as described by the following equations. Shear-thinning fluids extruded through a cylinder (Suntornnond et al., 2016) can be described by the following equations.

$$\tau_{w}=\frac{\Delta p\cdot R}{2\cdot L}$$ (3)

$$V_{avg}=\frac{n}{3n+1}{\frac{\Delta p\cdot R}{2k\cdot L}}^{\left(1/n\right)}\cdot R$$ (4)

$$Q=\pi R^{2}V_{avg}$$ (5)

R and L are the radius and length of the pipe, V_avg_ and Q are the average velocity and flow rate, k and n are the consistency coefficient and power-law index, respectively. With the input parameter combinations obtained from the DoE, CFD simulations were run for each case and the shear stress (τ), along with other useful parameters such as flow rate (Q), flow velocity (v) and viscosity profile (ν), was computed to understand the influence of the nozzle geometry on the printability and cell viability.

### Data Analysis

Analysis of the *in silico* data obtained from the CFD simulations was performed using the “R” (version 3.6.1) open-source statistical programming language with “RStudio” GUI/IDE (Ihaka et al., 1996; Allaire, 2012). To evaluate the importance of the input parameters, a machine learning algorithm involving a Gaussian process was utilized, implemented with the “tgp” package (version 2.4–14) (Gramacy, 2007). The “tgp” package creates a machine-learning model (Gaussian Process, functions: “bgp()” and “sens()”), which enables quantification and visualization of the sensitivity of each parameter towards the model response (maximum shear stress in our case), and the varying trend of the input parameters with the response variable. All the data wrangling and plotting were done with the package collection—“tidyverse” (version 1.3.0) (Wickham et al., 2019) and “ggplot2” (version 3.3.2) (Wickham, 2016), respectively.

## Results

### Shear Stress as Predictor of Cell Viability

**FIGURE 2:**
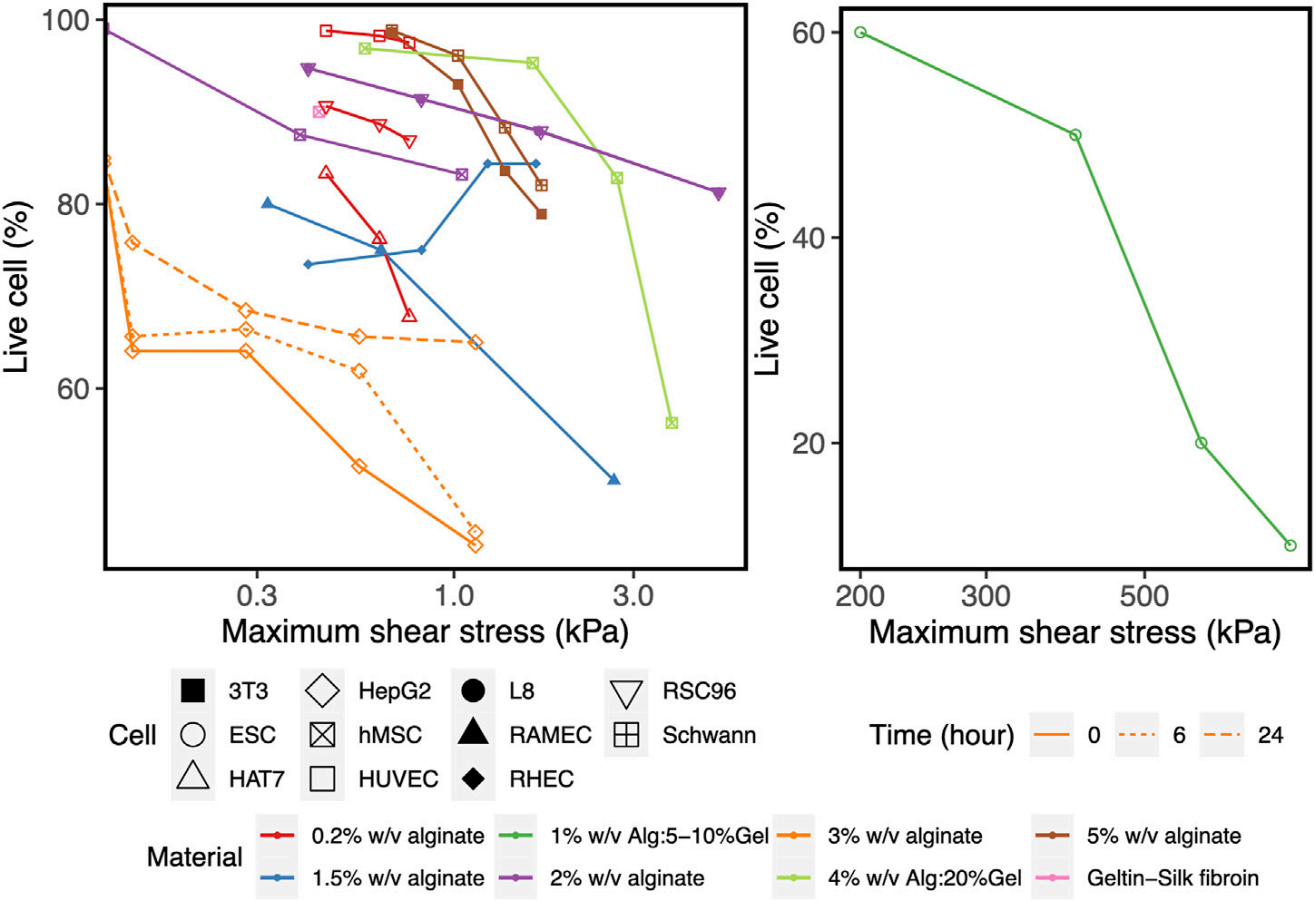
The effect of the maximum shear stress (quantified by the simulations) on cell viability (obtained from experiments reported in the literature). The cell and print settings of the experiments are presented in Table 3. They include Input parameters such as types of cells (point shapes), material properties (point and line colors), duration after printing (line types). Note: the X-axis is presented in log_10_ scale for better visibility. Cell viability at a shear stress of 0kPa indicates the value for the 2D control.

To corroborate the use of shear stress as a predictor for cell survival in 3D bioprinting, we simulated a series of experimental studies reporting cell viability results for a range of printing parameters such as pressure, nozzle radius, flow rate, material properties and duration after printing. The computed maximum shear stress value (MSS, maximum of τ) for each parameter combination (using our CFD simulation platform) is plotted against the experimentally measured cell viability (as reported in the literature) in Figure 2. Cell viability decreases significantly with increasing shear stress values, except for one experiment where cell viability remained more or less constant with the author reporting the absence of statistical significance (Khalil and Sun, 2009). All the chosen studies, used a blunted nozzle shape, facilitating the direct comparison. Whenever all the parameters are not found from the report (such as radius of nozzle entry or length of the lower nozzle in a few cases), it is assumed by comparing with similarly shaped nozzles in the NordonEFD bioprinting nozzle catalogue (Table 3). The exact relation between cell viability and shear stress depends on several variables, including the material and the cell type. Based on these results, in the remainder of this study, MSS is used as the output quantity to evaluate the different nozzle designs.

**TABLE 3:** Cell and print settings for studies investigating the effect of bioprinting conditions on cell survival. The maximum shear stress (MSS) is calculated by Computational Flow Dynamics (CFD) simulations in the present study, shown graphically in Figure 2. Some studies reported the shear stress and corresponding cell viability, but without all the parameters required to perform CFD simulation of the bioink in the nozzle (indicated by *).

Material	Pressure (kPa)	Nozzle diameter (Rs, mm)	Length lower (Ll, mm)	Flow rate (µL/s)	MSS (kPa)	Live cell (%)	Cell	References
3% w/v alginate, K: 8.587, *n* = 0.76	0–275.79	0.150–0.400	12	0.6–226	0–2.17	42.97–83.84	HepG2 (liver hepatocellular carcinoma cell)	Chang et al. (2008)
1.5% w/v alginate, K: 1.259, *n* = 0.7	55.12–220.48	0.250	12	0.4–1.6	0.41–1.65	73.44–84.38	RHEC (rat heart endothelial cell line)	Khalil and Sun, (2009)
1.5% w/v alginate, K: 1.259, *n* = 0.7	34.45–275.60	0.250	12	0.3–2.7	0.32–2.66	50–80	RAMEC (rat adrenal medulla endothelial cell)	Nair et al. (2009)
5% w/v alginate, K: 10.82, *n* = 0.54	200–500	0.250	20	2.3–12.6	0.68–1.708	78.91–98.83	3T3 (mouse embryonic fibroblast, “3-days transfer, inoculum 3×10^5^ cells”)	Li et al. (2011)
2% w/v alginate, K: 2, *n* = 0.87	0–275.60	0.150	12.70	0.1–0.3	0–1.05	83.2–99.02	hMSC (human mesenchymal stem cell)	Snyder et al. (2015)
1% w/v Alg:5–10%Gel,K: 8.183,*n* = 0.546	–	0.250	–	0.700	200–600	10–60	ESC (embryonic stem cell)	Ouyang et al. (2016)*
AlgGel, K = 13.3, *n* = 0.608	100.00	0.200	12.22	0.6–3.8	0.58–3.79	56.25–96.88	hMSC (human mesenchymal stem cell)	Paxton et al. (2017)
0.2% w/v alginate, K: 25.60, *n* = 0.35	50.00	0.200	6.35	1.5–197	0.41–5.05	81.25–94.73	L8 (rat myoblast cell), RSC96 (rat Schwann cell)	Ning et al., (2018)
2% w/v alginate, K: 10.39, *n* = 0.41	–	0.200	11	5–17	00.457–0.76	67.75–98.8	RSC96, L8, HUVEC (human umbilical vein endothelial cell)	Ning et al. (2020)
Geltin-Silk fibroin	–	0.200	6.35	–	0.440	90.00	hMSC (human mesenchymal stem cell)	Trucco et al. (2021)*

### Qualitative Description of the Response Variables

**FIGURE 3:**
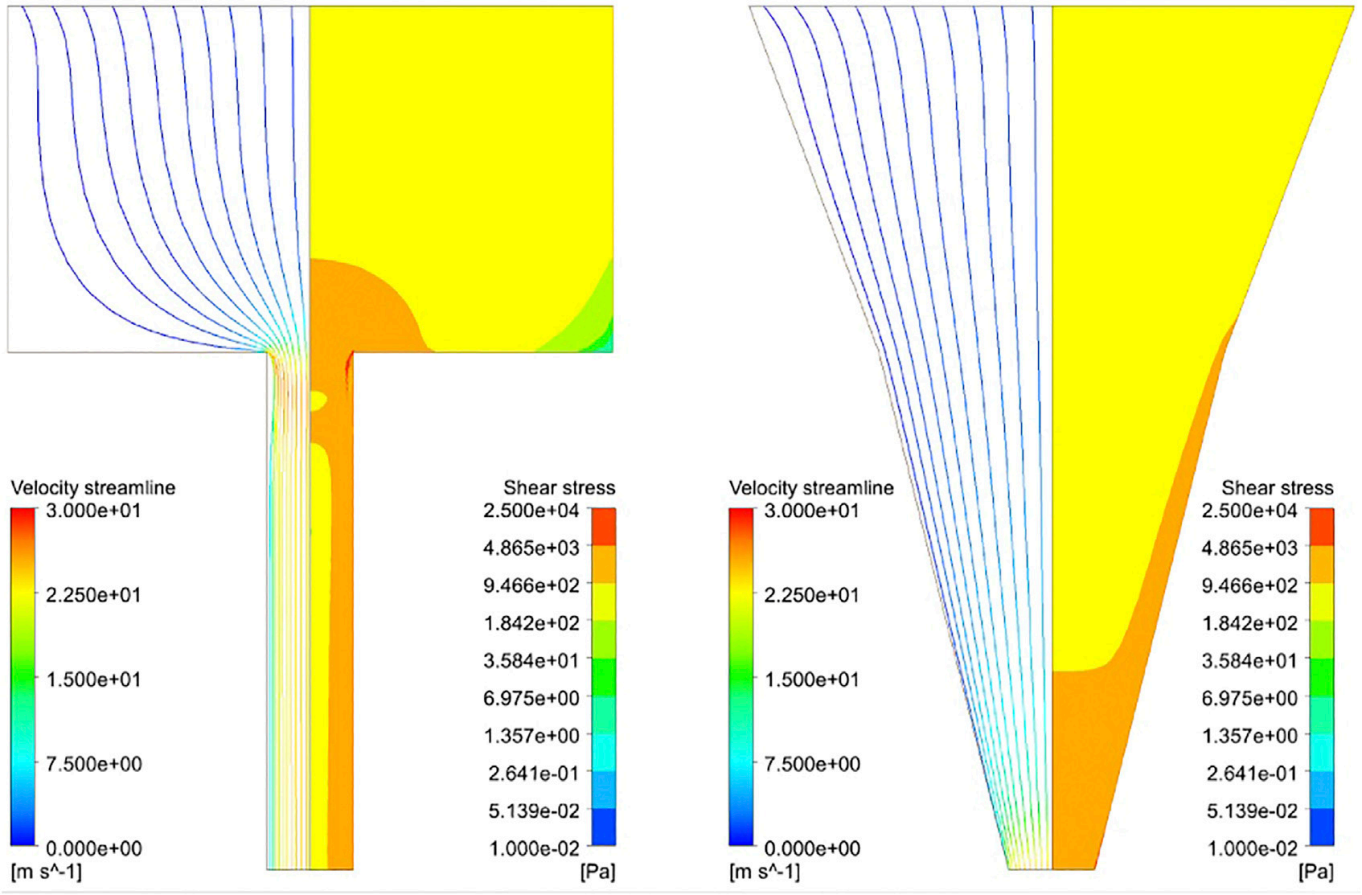
Streamline profile **(left part)** and shear stress distribution **(right part)** of Alginate hydrogel simulated at 340kPa inlet pressure with *R*_*big*_ = 3.5 mm, *R*_*small*_ = 0.5 mm, *L*_*upper*_ = 4.0 mm, *L*_*lower*_ = 6.0 mm and *R*_*middle*_ = 2.0 mm (*R*_*middle*_ for the conical nozzle only). Streamlines are colored according to velocity and the maximum shear stress zone developed in the hydrogel while printing (in red, 5–25 kPa).

Figure 3 shows the distribution of the shear stress throughout the blunted and the conical nozzles. This distribution is similar for all materials and nozzle design parameters, so only results for one material (Alginate) for both nozzle shapes are depicted. As expected, the shear stress presents a gradient with a low value at the inlet (<2 kPa), reaching its maximum value at lower part of the nozzle. Another important observation is that this maximum shear stress (MSS) occurs only at the nozzle outlet for the conical design, while a region of high shear is present throughout the whole lower nozzle for the blunted design.

**FIGURE 4:**
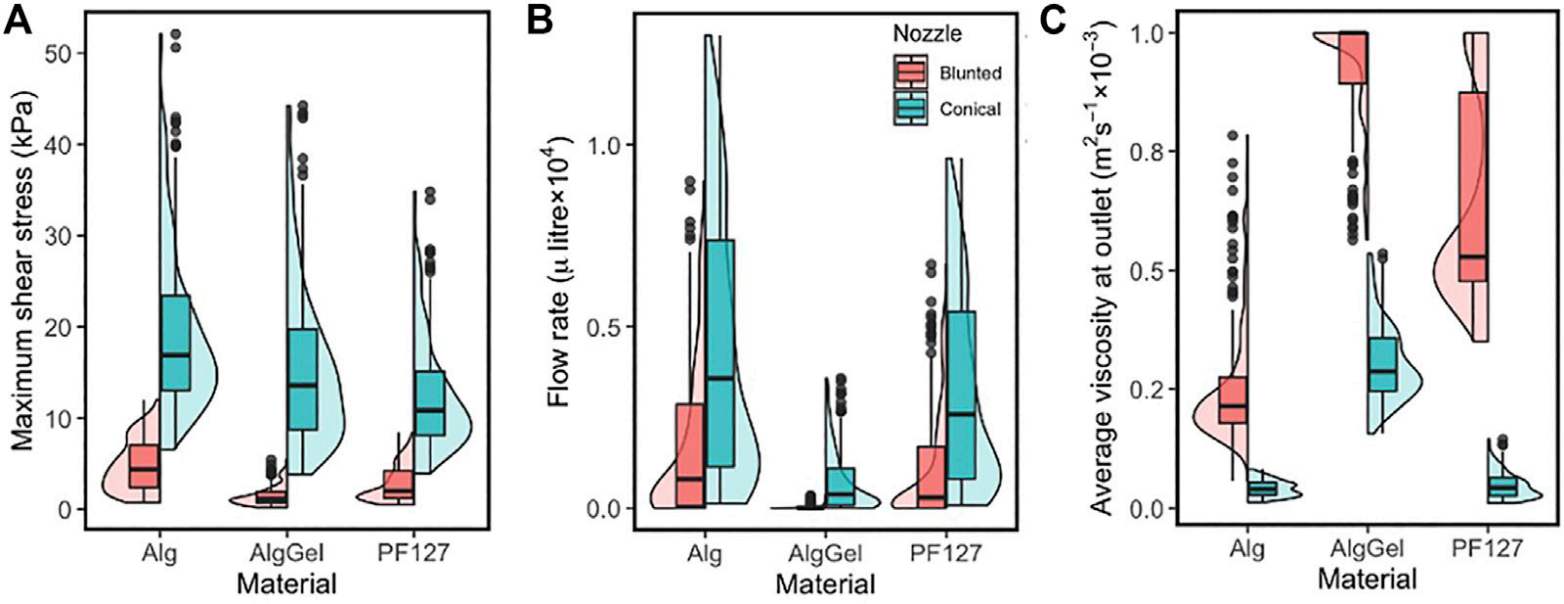
Combined box and violin plots summarising **(A)** the maximum shear stress; **(B)** the flow rate at the nozzle outlet and **(C)** average viscosity at the nozzle outlet—obtained from CFD simulations of 200 nozzle design for three different materials. Results are shown for the blunted nozzle (red, **left**) and the conical nozzle (green, **right**).

The box/violin plot of Figure 4 shows the distribution of important response variables such as the MSS (left), flow rate (middle) and the average viscosity of bioink material at the nozzle outlet (right) for all the 1,200 simulations (2 nozzle designs × 3 materials × 200 unique designs). Less variation in the MSS is observed for blunted nozzle designs than for conical nozzle designs in the investigated parameter ranges. For the conical nozzle, maximum shear stress ranges between 6.5–52, 3.8–44 and 4–35 kPa for Alginate, Alginate-Gelatin and PF127, respectively; and for the blunted nozzle those are 0.7–12, 0.2–5.5 and 0.5–8.5 kPa, respectively. Between the two types of nozzles, conical nozzles achieve higher maximum shear stress, but also yield higher flow rates (middle plot in Figure 4), meaning increased printing speeds compared to the blunted designs.

### Quantification and Trends Using Machine Learning

**FIGURE 5:**
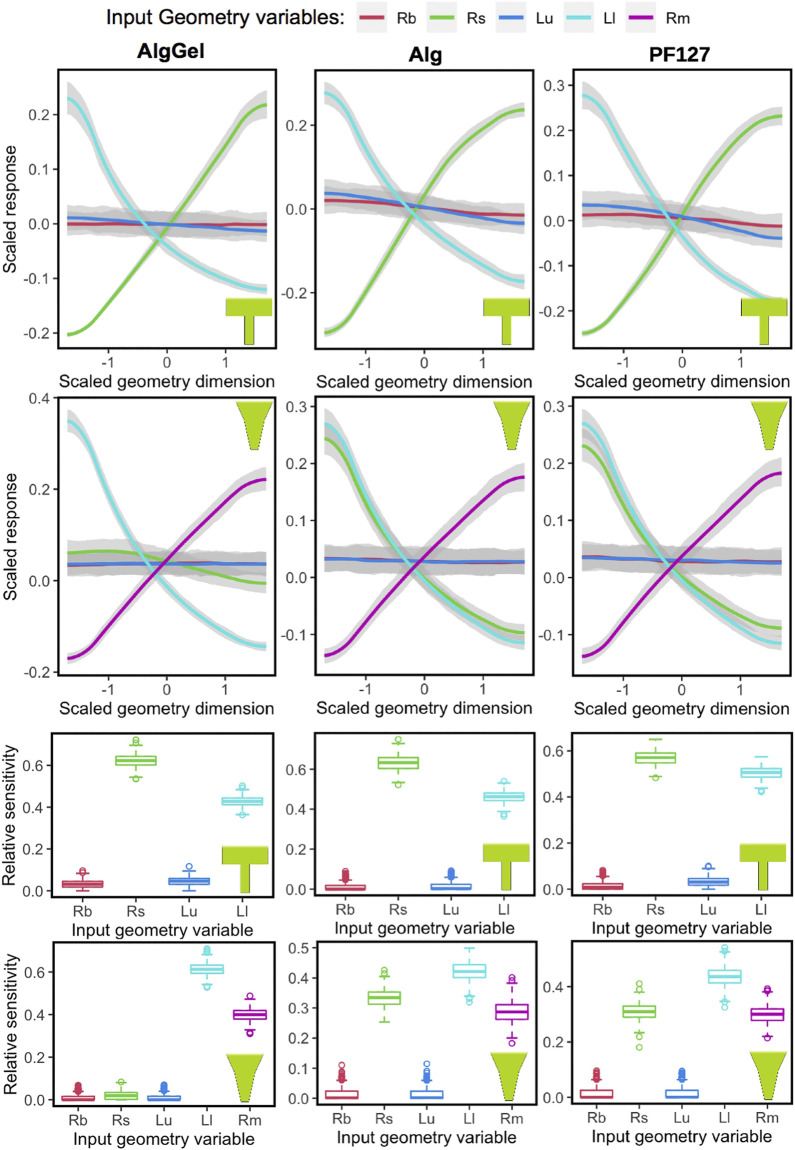
Influence of nozzle geometrical parameters (Rb = *R*_*big*_, Rs = *R*_*small*_, Lu = *L*_*upper*_, Ll = *L*_*lower*_ and Rm = *R*_*middle*_) on the maximum shear stress response, obtained using Gaussian Process, for different biomaterials (columns) and nozzle shapes (blunted or conical, in rows). The main effects are shown in the two top rows, where the “0” value in the X-axis represents the middle value from the nozzle geometry parameter range, and −1 and +1 are towards the low and high limit, respectively. Similarly, higher values of the scaled response (vertical axis) are associated with higher values of maximum shear stress. The sensitivity of each variable towards the model is summarized in the box-plots at the two bottom rows. Relative sensitivity at value “0” indicates no influence.

The Gaussian Process model captures the sensitivity of all parameters, which is evident from the 90% confidence intervals (the grey region around the trend-lines in two upper rows of Figure 5). Also, randomly dividing the dataset and running GP for each material-nozzle combination yields identical results, which validates its applicability to a new dataset (Supplementary Figure S2, S3). The plots of the two upper rows in Figure 5 show the influence of each geometrical parameter on the MSS for the three hydrogels modeled (Alginate-Gelatin, Alginate and PF127). The outputs are centered and scaled so that all parameters can be seen in a single graph to observe their relative influence on the response measure. When varying *R*_*big*_ and *L*_*upper*_ for either nozzle shape, the response remains unaltered (a flat line along the X-axis), indicating that the nozzle entrance radius and the upper nozzle length have a negligible influence on MSS. In contrast, *R*_*small*_, *L*_*lower*_ and *R*_*middle*_ (for conical only) have considerable influence on the MSS for both nozzle shapes, meaning that the geometry of the bottom part of the nozzle has most influence. The sensitivity of each geometrical parameter to the overall output variable MSS is shown in the box-plots at the bottom rows of Figure 5, with similar conclusions drawn from the main effect plots in the upper panels. For example, for a blunted nozzle shape combined with Alginate ink, *R*_*big*_ and *L*_*upper*_ having nearly no influence on the MSS; whereas the two other parameters, *R*_*small*_ and *L*_*lower*_, having altering values, contributes significantly to the MSS.

**FIGURE 6:**
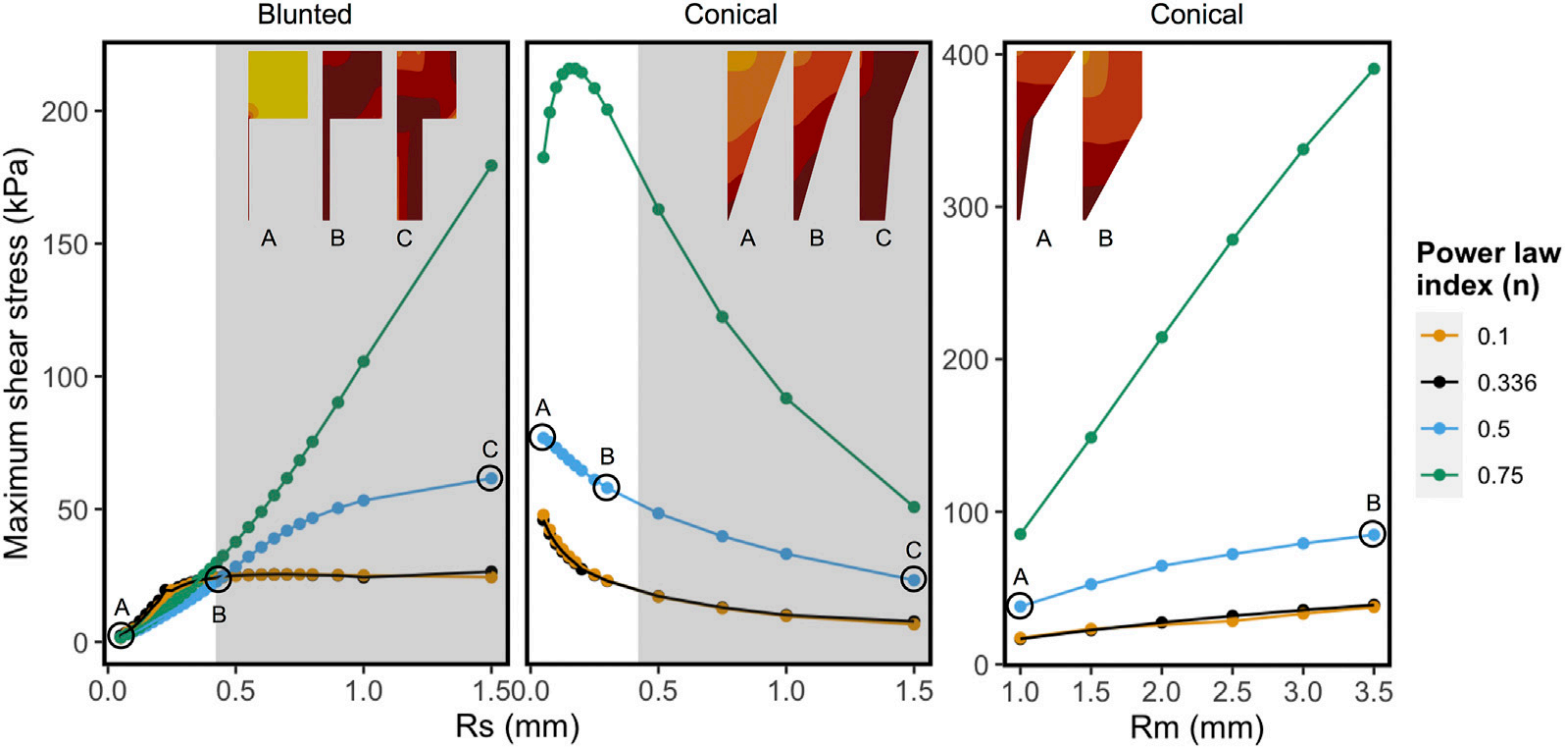
Influence of shear-thinning material properties on the maximum shear stress keeping all other factors constant for each panel. Every point and trend-line are colored with distinct values of “n” (power-law index). Low (*n* = 0.1) and high values (*n* = 0.75) represent highly shear thinning and almost Newtonian fluids, respectively. The left and the middle panels show maximum shear stress (MSS) in function of exit nozzle radii (Rs) at Rm = 2.0 mm. Right panel shows MSS in function of middle nozzle radii (Rm) at Rs = 0.2 mm. The nozzle shapes and relative MSS distributions are shown in the inset (darker colors correspond to relatively higher shear stress region, not to the same scale). The grey regions in the graphs with varying Rs correspond to the values that are beyond the range of commercially used 3D printing nozzles.

Figure 6 shows the combined effect of shear-thinning properties (power-law index, n) and the geometrical variables *R*_*small*_ and *R*_*middle*_ on the MSS for Alginate materials, while the other parameters are kept constant. Shear-thinning reduces the effect of geometric variation on the MSS. For the conical geometry, the effects of increasing *R*_*middle*_ on the maximum shear stress is linear for all values of “n”. In case of *R*_*small*_, the grey region on the right corresponds to values that are beyond the range of commercially used nozzles. *R*_*small*_ has opposite effects on MSS for the blunted and the conical design, as could already be observed in Figure 5. For almost Newtonian materials (*n* = 0.75) the conical nozzle shows more complex behavior with varying the power-law index and *R*_*small*_, where shear stress first increases sharply and then decreases for larger values of *R*_*small*_.

**FIGURE 7:**
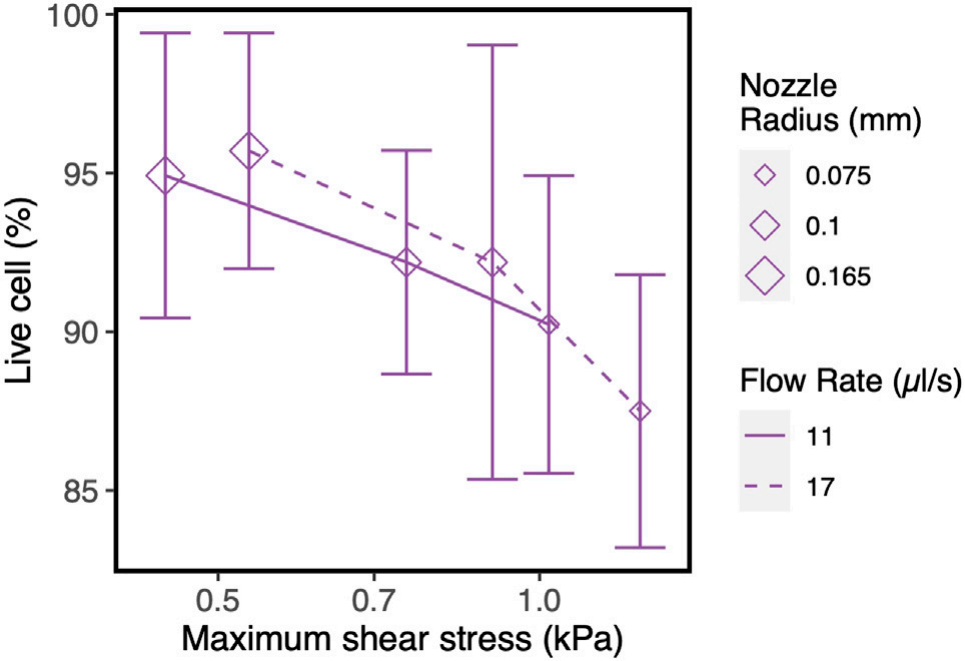
Influence of nozzle diameter on cell survival/recovery when printed at constant flow rate with varying nozzle diameter, vs. shear stress values obtained in our CFD simulations. The experimental results and model input parameters are taken from the reference (Ning et al., 2020).

The counter-intuitive results for the blunted geometry, where MSS increases for increasing values of the outflow radius, are a consequence of the chosen print strategy where a constant pressure difference is maintained for all nozzle designs during the print process. Table 4 contains the computed flow rates obtained during pressure-driven printing and vice-versa for a set of conical and blunted nozzles, where the *R*_*small*_ and *R*_*middle*_ values are varied within the commercially used range. This shows that in order to maintain a constant pressure difference during printing for the various nozzle designs, the flow rate significantly increases which causes an increase in MSS. When a flow-driven process is simulated for the same nozzle designs (through adaptation of print pressure), an increase in *R*_*small*_ results in a decrease in MSS, for both nozzle types. Ning et al. experimentally studied cell viability after printing at constant flowrate but with different nozzles diameter (Ning et al., 2020). The trend in viability they observed corresponds with the computational model predictions where smaller lower nozzle diameters (*R*_*small*_) led to lower maximal shear stresses and hence higher predicted viability (Figure 7).

**TABLE 4:** Quantification of the difference between pressure-driven and flow rate-driven bioprinting and their effects on response variables such as maximum shear stress, viscosity and velocity. Also, for the pressure-driven bioprinting, the resulting flow rate is quantified (last column). For flow rate-driven bioprinting the resulting pressure difference is quantified.

Input				Output			
Simulation	Nozzle	Rs	Rm	Max shear stress (kPa)	Viscosity (m^2^s^−1^)	Velocity (ms^−1^)	Flow rate (µLs^−1^)
Constant pressure (340 kPa)	Conical	0.227	1.000	14.39	4.60E-05	21.08	3,420.49
		0.227	3.500	34.73	2.69e-5	23.70	3,776.19
		0.050	1.729	39.67	1.32e-5	20.03	159.06
		0.420	1.729	15.54	5.99e-5	23.22	12,799.20
	Blunted	0.050	–	1.27	3.96e-4	0.07	0.57
		0.420	–	8.87	2.91e-4	15.10	8,355.76
**Simulation**	**Nozzle**	**Rs**	**Rm**	**Max shear stress (kPa)**	**Viscosity (m^2^s^−1^)**	**Velocity (ms^−1^)**	**Pressure (kPa)**
Constant flow-rate (3,500 µLs^−1^)	Conical	0.227	1.000	14.93	4.53e-5	21.66	360
		0.227	3.500	31.56	2.81e-5	22.06	300
		0.050	1.729	1,627.14	1e-5	441.65	100,000
		0.420	1.729	4.09	1.13e-4	6.33	530
	Blunted	0.050	–	955.74	1.2e-4	446.14	330,000
		0.420	–	5.09	1.2e-4	6.32	130

## Discussion

Extrusion-based bioprinting is at the heart of much research activity due to its potential use in a regenerative medicine context as the technology allows for the deposition of materials and cells in a 3D spatially controlled manner. The process still suffers from a low cell viability post-printing compared to other bioprinting modalities. Cells in the nozzle of a bioprinter can undergo cell death and unwanted cell differentiation due to (too high) shear stress. In this study, a computational model was used to study the effect of the nozzle geometrical parameters on the maximum shear stress, shear rate and outlet hydrogel velocity in a DoE approach. 200 designs each for both the blunted and the conical nozzles were built by varying geometrical parameters within a predefined range using LHS. In addition to the nozzle shape, different hydrogels (each with a different level of shear-thinning) were used during the simulations, resulting in a total of 1,200 simulated nozzle shape-biomaterial combinations tested under fixed printing pressure. After initial model validation, a series of screening experiments reported in the literature was modeled to corroborate the use of maximum shear stress as a measure of cell viability. The influence of geometrical parameters on MSS for the six cases was analyzed through Gaussian Processes and implications for the design of tailored nozzles were investigated. Finally, these computational results were compared to dedicated experiments reported in the literature investigating the effect of the lower nozzle diameter in blunted nozzles.

*In silico* models of extrusion-based bioprinting reported in the literature often reduce the model domain to the lower part of the nozzle, especially for the blunted nozzle (Khalil and Sun, 2009; Rezende et al., 2009; Lee and Yeong, 2015; Paxton et al., 2017). Such a simplified geometry is not capable of capturing the pressure drop in the upper part of the nozzle. Under pressure-driven flow conditions such as simulated in this study, we found that the pressure drop in the upper part of the nozzle might not always be negligible as assumed by some studies using analytical models, such as described by Eqs 3, 4. Even if “Δ*p*” is kept constant, it affects the flowrate with varying geometrical parameters, hence the printability and also the shear stress magnitude (see Table 4). In other words, the assumption can be valid in a few cases depending on the values of *Llower* or *Rsmall* but not over the entire range of commercially available 3D printing nozzles, which we can easily visualize from the pressure difference in our simulations. Such advantages encouraged the bioprinting community to use CFD based simulations in recent years (Billiet et al., 2014; Emmermacher et al., 2020). It is worthwhile to mention that *in silico* bioprinting studies employing CFD modeling often also make several assumptions, serving to reduce complexity. These assumptions, also made in the present study, pertain to the no-slip boundary condition and incompressible and laminar flow of the bioink. Another assumption is the implementation of hydrogel bioink as single-phase without considering the encapsulated cells as separate entities, for which it was shown that cells with a diameter less than 10% of the nozzle radius can be assumed to be macroscopically monophasic with the hydrogel (Emmermacher et al., 2020).

A multitude of studies has focused on the *in silico* aspects of the bioprinting process, with either printability or cell viability as the focal point. In an experimental study, Webb et al. aimed to optimize print parameters by using nozzle diameter and pressure as the surrogate to account for the shear stress and printability, since there is no direct measure of shear stress experimentally (Webb and Doyle, 2017). Billiet et al. found the critical areas of shear stress in their CFD model for blunted and tapered nozzle design (Billiet et al., 2014), which corroborates well with our findings. Emmermacher et al. used a CFD model that takes account of hydrogel material property, pressure and nozzle diameter, however focusing particularly on the conically shaped print nozzle (Emmermacher et al., 2020). A thorough comparison of the blunted and conical nozzle for similar print conditions has not been done, with some experimental studies reporting only differential cell viabilities with the two types of the nozzle. The application of machine learning in the design optimization of 3D extrusion-based bioprinting has been hinted at in perspective papers (Kim et al., 2019; Yu and Jiang, 2020) but has not yet been reported, to the best of our knowledge. In this study, we have shown that with constant print pressure certain geometrical parameters such as the radius of exit nozzle radius, the two nozzle shapes can behave in an opposite manner in the commercially available range (Figure 6, varying *R*_*small*_). It also indicates that if the shear-thinning ability of a bioink material is varied, a contrasting shear stress response might be obtained at the limits of Newtonian and non-Newtonian behavior, even with the same type of nozzle (Figure 6, conical nozzle varying *R*_*small*_). Here, the utility of the ML (GP) approach over conventional (linear) regression is justified, since a linear response is obtained when regression models are used in DoE, which may not be correct in more complex systems (Krausch et al., 2019).

Across all the nozzle geometrical parameters, all the parameters have consistent responses on the maximum shear stress, except *R*_*small*_. The main reason behind this is that for the pressure-driven bioprinting scenario, the flow rate does not remain constant with varying *R*_*small*_. A higher flow rate in the broader nozzle associates with higher flow velocity, which in turn leads to higher maximum shear stress in a blunted nozzle. The situation is more complex in conical, where higher flow rate indeed associates with increasing nozzle radius (conical nozzles at constant pressure in Table 3), however the shape of the lower nozzle changes (change in the tapering angle) unlike the blunted which affects the shear stress distribution. With the low *R*_*small*_, the tapering angle is large, and the stress is concentrated near the nozzle exit whereas for a high *R*_*small*_ the shear stress spreads to a larger area with less maximum value. Increasing *R*_*middle*_ (hence increasing the tapering angle) while keeping all other factors constant, leads to higher shear stress (Table 4). It might be helpful to think of the blunted nozzle as a simpler case of the conical, where *R*_*small*_ = *R*_*middle*_. We can see from Figure 5 that the trends in *R*_*small*_ in the blunted nozzle resemble those of *R*_*middle*_ in the conical nozzle. The highest shear stress region occurs near the nozzle tip in the conical but near the middle portion for the blunted nozzle. This is commensurate with what differentiates the two nozzle types, namely the shear stress distribution, as noted by some prior studies (Billiet et al., 2014; Paxton et al., 2017; Ning et al., 2020). Very few studies have reported comparing different nozzle geometries for extrusion-based bioprinting, however the number of geometries tested per study is limited (Li et al., 2011; Jiang et al., 2019; Emmermacher et al., 2020). Another asset of *in silico* modeling is that it provides a common platform to quantify and compare different experimental studies as shown in Figure 2, thereby allowing to aggregate insights from different studies.

Not only different geometries can be compared through a quantitative *in silico* model platform, but also simulated results from studies executed with different bioinks can be quantitatively compared. It is reported that the Non-Newtonian behavior of hydrogels is only strong with power-law indices below 0.5 and polymer behaves almost as a Newtonian fluid at values of 0.8 and higher (Chhabra and Richardson, 2008). This study showed that the shear-thinning capacity (high vs. low “n”) of material results in the reduction of the shear stress in the nozzle. Varying the composition for composite hydrogels such as AlgGel has been shown to affect both the printability and cell viability, in addition to the change in other processing parameters like gelation temperature, holding time, incubation time (Ouyang et al., 2016). For the blunted geometry, at all levels of shear-thinning, increasing the *R*_*small*_ at first leads to higher MSS, with decreasing effect as *R*_*small*_ approaches *R*_*big*_. However, the extent of shear-thinning yields contrasting effects in the conical nozzle. As mentioned before, varying *R*_*small*_ leads to the change in two factors simultaneously: the shear stress distribution and the flow rate, which oppositely affect the MSS. For more Newtonian material (*n* = 0.75), an increase in flow rate dominates first, leading to an increase in MSS, but then the change in the shear stress distribution starts dominating and MSS decreases, leading to a relatively complex response with an inflection point, for increasing *R*_*small*_ in the conical nozzle. It can be envisaged that with a certain combination of material properties and geometrical parameters (*R*_*small*_), these two factors can even equalize each other, leading to almost no influence on the maximum shear stress. This is observed in our simulation with a low shear-thinning material (AlgGel, *n* = 0.608) in the conical nozzle (main effect plot in Figure 5). This also substantiates the importance of the interaction between material property and nozzle geometry in a particular range of values relevant to 3D bioprinting, to the shear stress experienced by cells.

Apartfrom printability, cell viability is the key aspect in evaluating the performance of a 3D bioprinted scaffold or construct (Sharma et al., 2020). However, no consensus has been reached on the relation between the influential parameters and cell viability. For example, shear stress has been shown to affect the cells adversely in many studies (Nair et al., 2009; Li et al., 2011; Billiet et al., 2014; Wüst et al., 2015; Paxton et al., 2017), whereas some studies report little or no influence (Khalil and Sun, 2009). The reason for this can be of methodological or of intrinsic nature. Firstly, the time point when reporting cell viability ranges from immediate post-printing to a much longer timescale, e.g., up to 7 days (Blaeser et al., 2016). Secondly, different cell lines are used in the literature to perform cell viability evaluation, each having inherently different abilities to recover and regain viability as reported by Chang et al. (Chang et al., 2008). Combining these elements with the different materials and print settings leads to the situation where direct comparison of the different studies is neither feasible nor advisable. Figure 3 and (Table 3) show that, given time, HepG2 cells recover and increase viability after printing with 3% w/v alginate bioink, as can be appreciated by comparing cell survival immediately after printing (orange regular line) and the situation 6 and 24 h after printing. Printing three different cell lines (HUVEC, RSC, HAT7) with 0.2% w/v alginate as bioink (red lines) resulted in different degrees of cell viability (Ning et al., 2020). However, L8 and RSC96 cells bioprinted with 2% w/v alginate as bioink (purple lines) showed no discernible difference in viability at the same printing condition (Ning et al., 2018). As an intermediate conclusion, almost all studies we encountered utilizing stem cells (such as hMSC, hiPSC, hESC) report the adverse effect of increasing pressure or shear stress on cell viability (Faulkner-Jones et al., 2015; Blaeser et al., 2016; Paxton et al., 2017; Emmermacher et al., 2020) (see Figure 2). In line with this, here we did not assume any particular cell-specific property, we focused instead on print conditions and geometrical parameters defining the physical forces such as maximum shear stress acting on the cells during printing, which affects cell viability (Sharma et al., 2020). If we compare the range of shear stresses reported in the literature using the analytical equations (up to 3 kPa, Figure 2, Eq. 3) with that obtained in our simulations (up to ∼50 kPa, Figure 4), the analytical equations yield a single value rather than the shear stress distribution obtained in the CFD simulations.

Increasing the nozzle diameter maintaining the same flow rate led to lower maximal shear stresses in the computational model (Figure 7), a trend confirmed by the experimental results. However, due to the particular combination of cell, material, nozzle and printing conditions, the experimental cell viability results did not result were not statistically significant. While for the general studies varying pressure or flow rate without altering the nozzle geometry we see a strong concordance between maximum shear stress and cell viability (Figure 2), the numerical and experimental results for studies with varying *R*_*small*_ for the blunted nozzles indicate that other factors might also be important in addition to the magnitude of shear stress. Residence time has been suggested by a number of studies as another strongly influential factor next to (or in some cases more important than) the magnitude of the shear stress (Li et al., 2011; Faulkner-Jones et al., 2015; Paxton et al., 2017; Ning et al., 2020). It is not only the magnitude of the shear stress but also the exposure time of the cells to that magnitude that will determine the extent of cell damage.

This is an area where we can build upon our present simulation platform to build a model that accounts both for the effect of residence time as well as shear stress on cells. Such a model could be used in a multi-objective optimization finding the printing conditions leading to a compromise between lowering shear stresses and increasing residence time. This is likely to provide further insight into the nature of the 3D printing process with shear-thinning bioink materials. Lastly, the approach presented here is not limited to nozzle geometrical parameters but is also applicable to other optimization aspects of bioprinting. One such scenario can be to obtain parameters for the fastest printing (using the flow rate as the response variable in the Gaussian Process model) while maintaining a predefined minimum level of cell viability.

## Conclusion

In summary, the findings demonstrate that shear stress and thus cell viability will be strongly influenced by specific geometrical features of the nozzle and the hydrogel selected in the bioprinting process, alone or in combination. The most influencing geometry parameters dictating the maximum shear stress, are the radius of the middle and the exit of the nozzle, in addition to the lower nozzle length. The effect of nozzle geometry and material properties (extent of shear-thinning) are not always separable, but rather influence the shear stress response along with other flow properties in a combinatorial way. The identification of crucial parameters that affect the cell fate or the flow properties and the relation between them, is the main benefit of introducing *in silico* modeling combined with a design of experiments approach, analyzed using machine learning methods, allowing to probe the whole commercially available print nozzle spectrum. This may reduce the number of preliminary trial-error experiments that need to be carried out and result in a reduction of the associated time and expenses. An additional advantage of *in silico* modelling is that it allows to quantitatively compare different experimental setups and their outcomes. We believe that the findings discussed here can stimulate further development and potential application of *in silico* tools in the relevant areas of the bioprinting field.

## Data Availability

The original contributions presented in the study are included in the article/Supplementary Material, further inquiries can be directed to the corresponding author.
