# Supplementary material for: Towards the Experimentally-Informed In Silico Nozzle Design Optimization for Extrusion-Based Bioprinting of Shear-Thinning Hydrogels
Source: Front Bioeng Biotechnol. 2021 Aug 6;9:701778. doi: 10.3389/fbioe.2021.701778 (PMC8378215; doi:10.3389/fbioe.2021.701778)
Supplement: Supplementary file 1 [file Table1.DOCX]

Supplementary Material

Towards the *experimentally-informed in silico* nozzle design optimization for extrusion-based bioprinting of shear-thinning hydrogels

Esther Reina-Romo, Sourav Mandal, Paulo Amorim, Veerle Bloemen, Eleonora Ferraris, Liesbet Geris

**1. Rheological curves for the hydrogel materials studied in the present study**


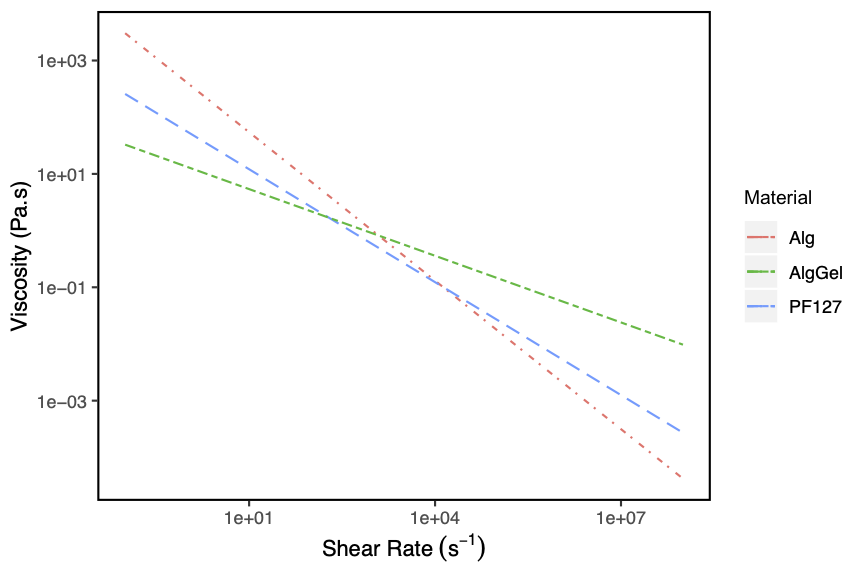


Figure S1: The shear-thinning nature of the three hydrogels are represented with the change in viscosity with shear rate (note the log scale).

# 2. Testing the validity of Gaussian Process model with random sampling data

The validation of any machine learning model can be done using various methods. One common approach is dividing the dataset into arbitrary parts and running the model individually with them and then ascertain whether they behave similarly. This enables the model to be used in similar but new conditions, in the range of parameters which was used to build the model. We have divided our 200 design points (or individual simulations) for each material-nozzle combination into two equal sized groups, by arbitrarily taken samples from the simulation dataset. They indicate almost identical behavior (see Figures S2 and S3, for the blunted and the conical nozzles respectively), to the parent model presented in the main manuscript. This provides confidence in the predictive power of the model – to be valid for a random set of bioprinting nozzle parameters for bioink material with defined rheology, print pressure and nozzle type. Also, the high precision (the grey region denotes the 90% confidence interval of the trend-lines) associated with the GP model informs us, which parameters can be safely ignored during optimization of the printing process.


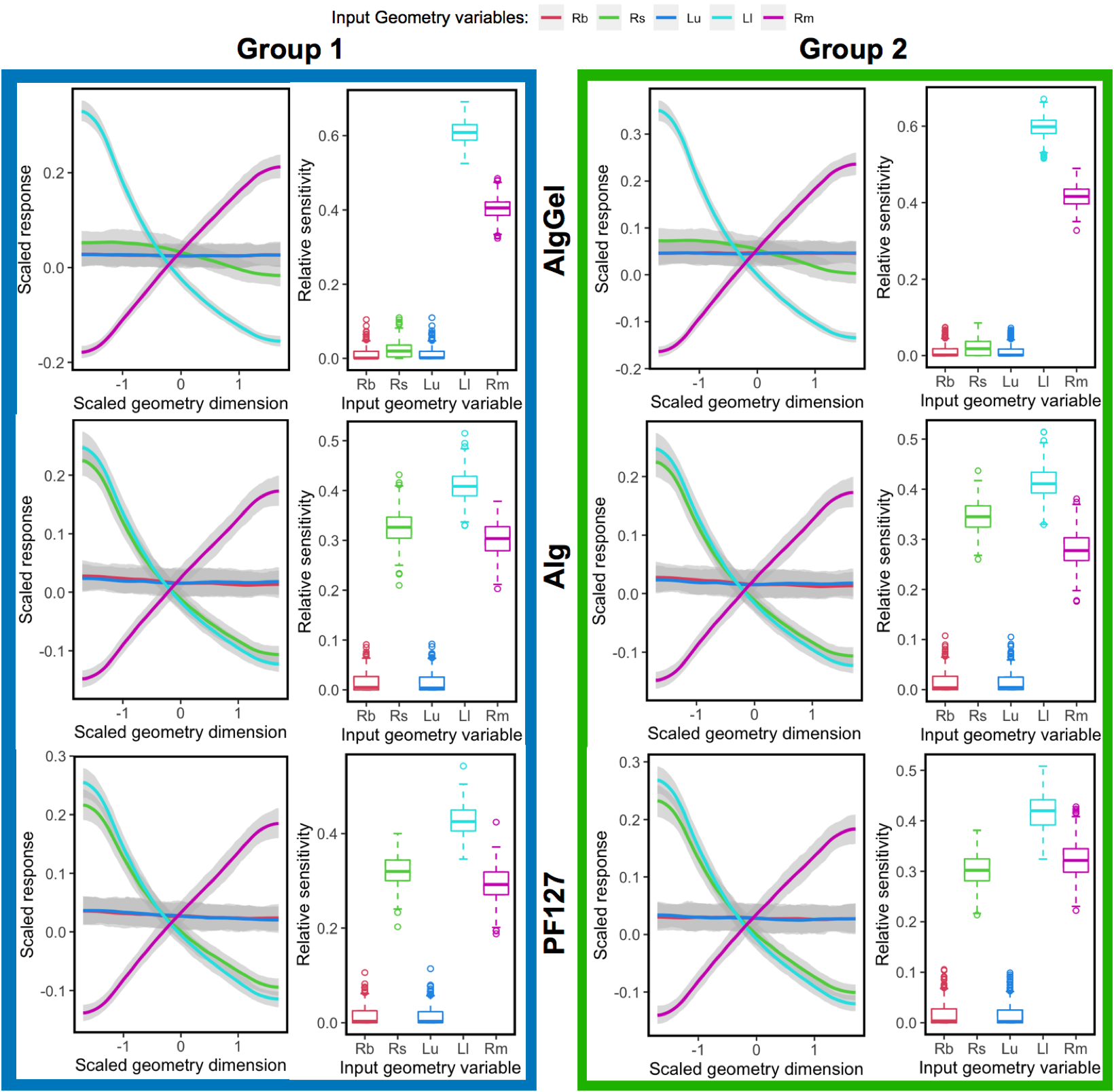


Figure S2: Gaussian process model on two equal sized groups with arbitrary sampling for each material, for the blunted nozzle design, provides identical response of the GP model for either group.


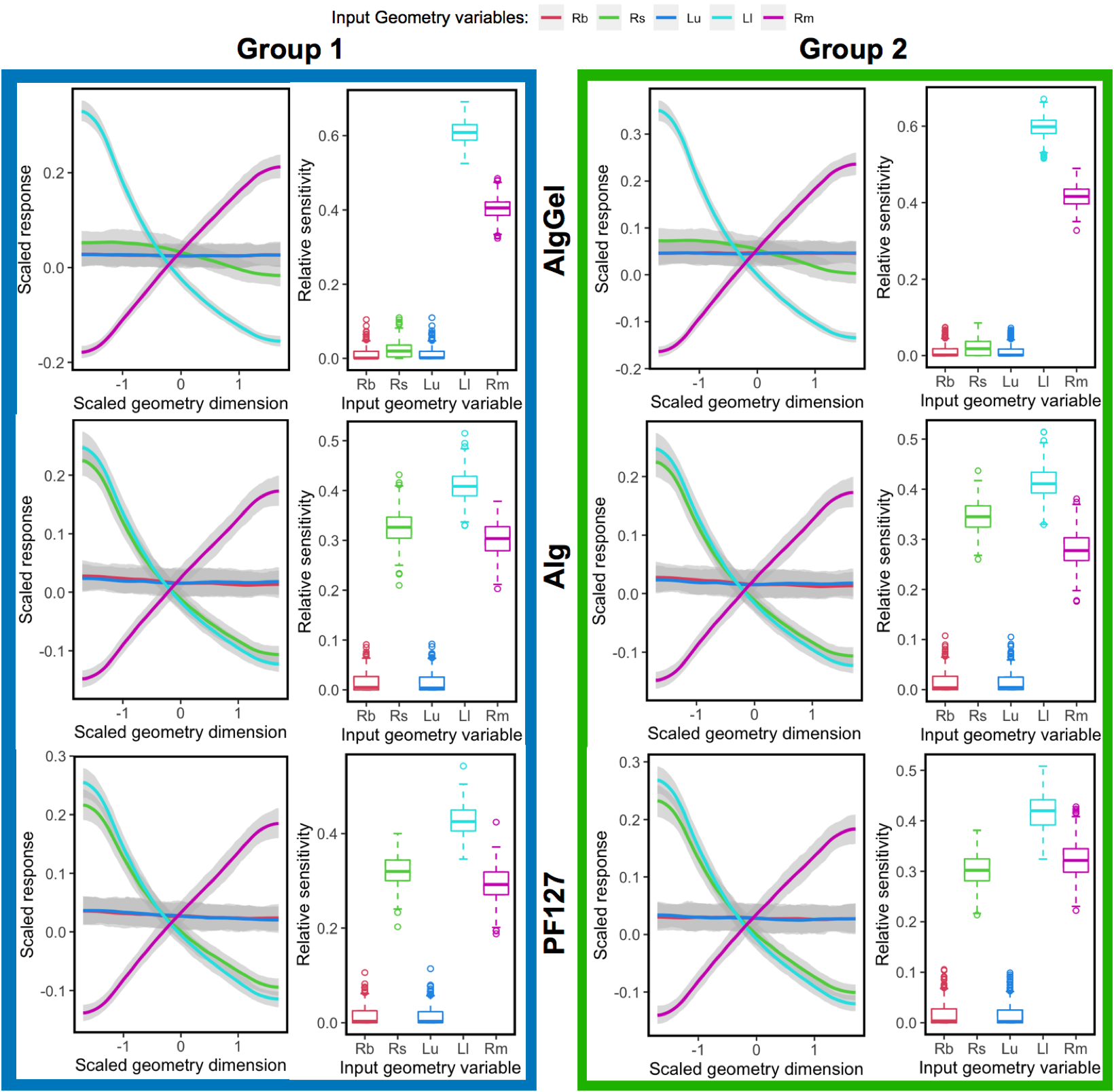


Figure S3: Gaussian process model on two equal sized groups with arbitrary sampling for each material, for the conical nozzle design, provides identical response of the GP model for either group.
